# Supplementary material for: Robust automated reading of the skin prick test via 3D imaging and parametric surface fitting
Source: PLoS One. 2019 Oct 21;14(10):e0223623. doi: 10.1371/journal.pone.0223623 (PMC6802838; doi:10.1371/journal.pone.0223623)
Supplement: Attachment — Submitted filename: PONE-response-letter.pdf [file pone.0223623.s001.pdf]

Dear Dr. Grisan,

We are submitting our response letter to the reviewer comments and the revised manuscript. Overall, we appreciate the constructive review, which has led to an improved paper. We want to make the peer review publicly available. Also, we have made the data and the analysis code available on a public repository. The conventions we have used in this letter are:

- Black text in italics are editor or reviewer comments
- Blue text are our responses to those comments
- Red text are the new or modified text in the manuscript. When necessary we have included a figure from the paper here or a screenshot with equations.

A redlined version of the manuscript is included in the new submission.

Regards,

Andrés Marrugo, Ph.D.
Associate Professor
Universidad Tecnológica de Bolívar
Cartagena, Colombia

---

a) If there are ethical or legal restrictions on sharing a de-identified data set, please explain them in detail (e.g., data contain potentially sensitive information, data are owned by a third-party organization, etc.) and who has imposed them (e.g., an ethics committee). Please also provide contact information for a data access committee, ethics committee, or other institutional body to which data requests may be sent.

b) If there are no restrictions, please upload the minimal anonymized data set necessary to replicate your study findings as either Supporting Information files or to a stable, public repository and provide us with the relevant URLs, DOIs, or accession numbers. For a list of acceptable repositories, please see http://journals.plos.org/plosone/s/data-availability#loc-recommended-repositories.

We have added the following statement on data availability to the manuscript which states the following:

Data availability

The authors confirm that all data underlying the findings are fully available without restriction. The final dataset and accompanying code are available on the Open Science Framework, link for peer review (the repository will be made public with DOI upon acceptance) https://osf.io/yk8u5/?view_only=14bfd4e0f19d4079872de4f5ce119535.

Reviewer #1

In my opinion, there are two serious objections about the technical quality of this work.

1) The authors claim that, to the best of their knowledge, this is the first time such a model (a parametric model to characterizing the 3D data of the wheals) is proposed (contribution in page 3). This model is shown in equation (1), that use Gaussian functions with a flat-top. In principle, the model sounds well, but if it the first time that a model is proposed, the authors should justify better the selection of the model. For example, take three or four possible models and test which one achieves better fit.

Related to this issue, I would like to know if Fig. 3 shows real results of the proposed method. I mean, if you compare the images of S'_w1, S'_w2, etc. with f_1, f_2, etc., the obtained model does not represent well the actual wheal. The shape of the "base" of the resulting model seems to be rectangular and I would like to use a model that achieves a kind of elliptical base. The use of Gaussian functions could achieve this, but I think that the use of super-Gaussian functions or Gaussian functions with a flat-top provokes that the base of the modeled wheal is nearly rectangular. In my opinion, it has no sense to model very precisely the high of the wheal but the base of the wheal (moreover if you want to obtain the maximum diameter of this base). Maybe a kind of cylindrical function, using an ellipse for the base and a constant high is a better model for this purpose.

Thus, if proposing a model is one of the main contributions of the paper, I would like to discuss better the goodness of the model.

RE: Thank you for your comment and for identifying an aspect that is worth discussing in more detail. It has allowed us to improve the quality of the manuscript.

As you mention, it is not extremely important to model the height of the wheal accurately, or for that matter, the overall 3D shape of the wheal. While this accurate modeling could be achieved with splines or other types of parametric surfaces, our aim is to fit a parametric model that automatically estimates a clinically relevant parameter. This parameter should be readily interpretable and comparable to the conventional reading of the SPT, which is why we have used the longest diameter. In other words, with our parametric model we are trying to mimic computationally what the physician does with a ruler, but doing so in a way that produces accurate and reliable results every time the same wheal is measured. We have avoided more complex segmentation algorithms, such as active contours because they are typically more computationally demanding, and are prone to noise or other artifacts.

To this end, we have rearranged our manuscript to describe a general parametric fitting framework in which we test two parametric models inspired by the conventional methods for measuring wheals. These approximations have been described in the introduction and illustrated with the new figure shown in Fig. 1. The new figure with its caption is the following:

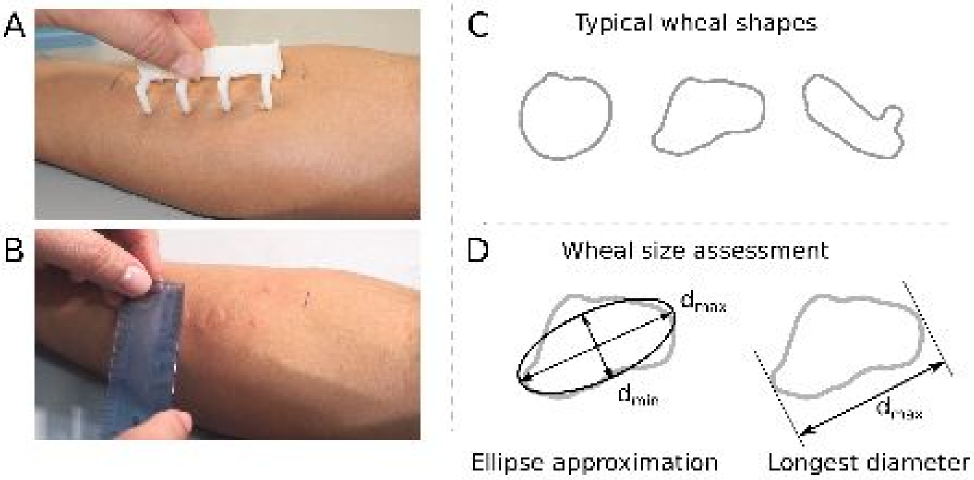

Fig 1. **(A)** The physician performs the SPTs with a disposable multi-test on the surface of the forearm. **(B)** After 15 minutes, the physician measures the skin reactions or wheals using a ruler. **(C)** Typical shapes of wheals, from regular to irregular shape with pseudopod. **(D)** The wheal size assessment is approximated as an ellipse or by measuring the longest diameter.

We defined two parametric models. The parametric model 1 attempts to measure the wheal with an inverted Elliptical Paraboloid function. This model yields an elliptical base from which the longest diameter is determined. In the parametric model 2, we use the super-Gaussian to approximate the longest wheal diameter as the longest dimension of a bounding box. Super-Gaussians have been used to model laser beams and determine its width. Also, if a distribution is, in fact, Gaussian, then the fitting should yield a Gaussian distribution. However, the parametric fitting results in a higher-order Gaussian distribution with a rounded rectangular base resembling a bounding box which contains the 3D distribution of the wheal. We have illustrated the difference of the two models with a new figure shown in Fig. 8 with the results of fitting a regular shaped wheal and an irregular shaped wheal. The figure is the following:

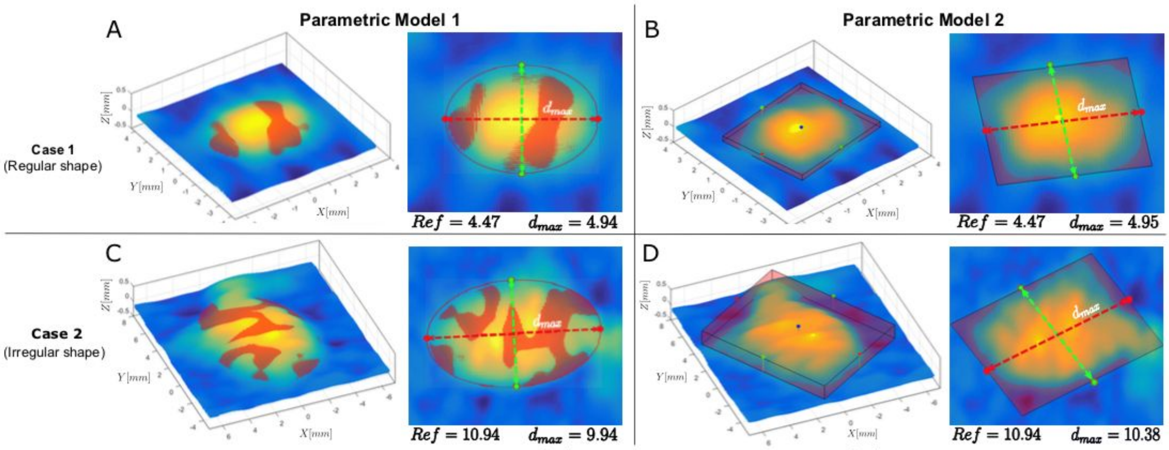

Fig 8. The results of fitting a regular-shaped wheal with (**A**) the *Parametric model 1* and (**B**) the *Parametric model 2*. Both models are in numerical agreement and slightly overestimate the reference measurement of 4.47 mm. For an irregular-shaped wheal the result from (**C**) *Parametric model 1* and (**D**) *Parametric model 2* differ by 0.44 mm. However, the result from *Parametric model 2* results in a more accurate estimation of the longest diameter.

We analyzed the accuracy of the two models against manual reference measurements from the physician. We also evaluated the ratio of the estimated diameters by the two parametric models, and it was close to 1, exactly 0.99 ± 0.022 or (0.97, 1.01) with 99% confidence. The CV of the computed ratios was 6.40%, which exhibits high reproducibility between the estimations from both methods. However, the parametric model 2 on average yields diameters closer to the reference measurements (model 1: $\bar{d}$ = -0.398 mm vs. model 2: $\bar{d}$ = -0.339 mm) with narrower 95 % limits of agreement (model 1: [-1.58, 0.78] mm vs. model 2: [-1.39, 0.71] mm) in a Bland-Altman analysis. For this reason, we considered the parametric model 2 as our candidate de facto model for performing the wheal diameter estimation in our system, and the one chosen for graphical representation in Fig. 9.

The robust diameter estimation section was rewritten to describe a general parametric fitting framework with the two parametric models. The section is the following:

Robust diameter estimation via parametric fitting

The purpose of this stage is to fit a parametric model that automatically estimates a clinically relevant parameter that is readily interpretable and comparable to the standard reading of the SPT. In other words, we are trying to mimic computationally what the physician does with a ruler, but doing so in a way that produces accurate and consistent results every time the same wheal is measured.

We define a general parametric fitting framework, and we test two parametric models inspired by the two conventional wheal measurement procedures described in the introduction. With the parametric model 1, we attempt to reproduce the shape of the wheal with an inverted Elliptical Paraboloid function, such that when evaluated at $z=0$ yields an ellipse. The major axis of the ellipse is adopted as the longest wheal diameter. With the parametric model 2, we approximate the longest wheal diameter as the longest dimension of a bounding box. Bounding boxes of probabilistic nature or confidence score have been massively used for object detection algorithms [34], and we believe this is a reasonable approach since wheals often have irregular shapes. Furthermore, since wheals have been reported as having a relatively flat top [19] and decreasing smoothly, we use a generalized version of the Gaussian function called the super-Gaussian which has more degrees of freedom. The super-Gaussian distribution has been used to model the intensity distribution of laser beams and determining its width [35], or for determining the size of micro-aneurysms [36], and other distributions [37].

These are the three stages of the general parametric fitting framework:

The parametric fitting stage consists of three steps:

1. For each surface S'_{w_i}, we set the origin at the center of mass to obtain S''_{w_i} by means of PCA [29].
2. From S''_{w_i}, we fit either of the two parametric models to the 3D surface data. To incorporate a rotational degree of freedom, the fitting is carried out in an (x', y')-coordinate system rotated clockwise about an angle θ given by

$$\begin{bmatrix} x' \\ y' \end{bmatrix} = \begin{bmatrix} \cos(\theta) & -\sin(\theta) \\ \sin(\theta) & \cos(\theta) \end{bmatrix} \begin{bmatrix} x \\ y \end{bmatrix} \quad . \tag{1}$$

   The angle θ now becomes an additional parameter to be estimated as part of each model. The parametric model optimization is solved with the Trust-region reflective non-linear least-squares algorithm [38].
3. Once the optimization is complete, the obtained relevant parameter per model yields the estimation of the wheal longest diameter.

These are the equations for parametric model 1:

Parametric model 1

The *parametric model 1* is based on an inverted Elliptical Parabaloid function, given by,

$$f_1(x', y') = -\frac{1}{c}\left(\frac{\left(x' - x'_0\right)^2}{w^2_{x'}} + \frac{\left(y' - y'_0\right)^2}{w^2_{y'}}\right) + \beta \ , \tag{2}$$

where $w_{x'}$ and $w_{y'}$ model the width of the wheal in x'- and y'-dimension, respectively; β is the surface offset; and c is parameter modeling the wheal height. A mathematical expression for the diameter of the wheal is obtained by evaluating $f_1 = 0$, as follows,

$$\left(\frac{\left(x' - x'_0\right)^2}{a^2} + \frac{\left(y' - y'_0\right)^2}{b^2}\right) = 1 \ , \tag{3}$$

where $a = \sqrt{\beta c w^2_{x'}}$ and $b = \sqrt{\beta c w^2_{y'}}$ are the relevant parameters for this model. Finally, the diameter d_i is computed as $d_i = \max\{2a, 2b\}$, i.e., the longest diameter, as is customary for the standard skin prick test reading [9].

And for parametric model 2:

Parametric model 2

In the *parametric model 2*, the skin wheals are modeled as 2D Super-Gaussian functions, given by,

$$f_2(x', y') = \gamma * \exp\left(-\left(\frac{\left(x' - x'_0\right)^2}{2\sigma^2_{x'}}\right)^{\beta_{x'}} - \left(\frac{\left(y' - y'_0\right)^2}{2\sigma^2_{y'}}\right)^{\beta_{y'}}\right), \qquad (4)$$

where γ is the parameter modeling the wheal height; $\beta_{x'}$ and $\beta_{y'}$ model the wheal flatness in x'- and y'-dimension, respectively; $\sigma_{x'}$ and $\sigma_{y'}$ are the relevant parameters modeling the width of the wheal in x'- and y'-dimension, respectively. By fitting Eq. (4) to S''_{w_i}, the measured diameter d_i is computed as $d_i = \max\{4\sigma_{x'_i}, 4\sigma_{y'_i}\}$, which exhibits good agreement with the spatial distribution of the wheal.

Since the parametric fitting is an integral part of our manuscript, we have updated the abstract, the introduction, the results, discussion, and conclusions regarding the parametric fitting and the results from the two methods. The reviewer can check the modified sentences in the redlined version of the paper. The reviewer can verify that including a model that approximates the wheal as an ellipse produces similar results as the super-gaussian model on the estimation of the longest diameter. We chose the model that, on average, performs better.

2) The authors admit in page 5 that S' includes bumps due to wheals but also pores, follicles, wrinkles and hair (that are not wheals but the algorithm could see as wheals). However, in page 6 section wheal detection, they assume that applying a blob detector the wheals are automatically detected, and cite reference [27]. Are you completely sure that you are not obtained false wheals? The authors mention in the introduction, page 2, that a previous work has coped with false positives [13]. This issue should be better discussed. In fact, I'm not very convinced that the 11 wheals not detected by the physician through visual inspection are real wheals (page 9). Moreover, Table 3 reports real wheal for both the positive control (histamine) and the negative control (diluent !). For the positive control, a wheal is expected… but, for the negative control no wheal should arise!

Read please again the sentence "Note that for two wheals (cat dander and negative control) the physician assigned a negative reaction (no wheal) probably because the wheal was very small" (page 11). It is very strange. Probably the physician is right, and at least with the negative control (diluent) there was no wheal (and the pretended wheal reported in Table 3 is a false positive). The authors must clarify this point.

RE: Thank you for your comment and for identifying an issue that may generate confusion on the reader. We are confident of the wheal detection results because of the multi-test pricking device that we used for our experiments; this was not the case in the work by Justo et al., 2008. We have now included a new figure (Fig. 1), in which we illustrate better how we performed the SPT. It is certainly visible from the resulting wheals that their scale is different from follicles, pores, and other skin features of sub-millimeter size. This aspect of the filtered

3D reconstruction is also evidenced in Fig. 6 in which we have included the texture image to confirm the detections. Also, the regularly distributed wheals arise from the multi-test device, we verified that all detected wheal locations were consistent with the multi-test pattern. Although our algorithm currently does not enforce wheal detection locations following a specific pattern, in future work we may include this feature, especially when using multi-tests with more than eight pricking sites.

Moreover, false-positive reactions on the negative control site are known to occur due to the trauma that the pricking device imparts on the skin. This situation is also dependent on the skin sensitivity of the subject. In some cases, a large wheal on the Negative control site is an indication of a condition called dermographism urticaria, which invalidates any diagnosis produced from the SPT due to the possibility that a wheal on another site was due to trauma and not an allergic reaction. Nevertheless, this issue does not invalidate the wheal measurements from our device; it is the physician who has to interpret the measurements. To clarify your raised concern in the manuscript, we have modified the sentence reporting the results for experiment 2 as follows:

Note that for two wheals (C. dander and N. control) the physician assigned a negative reaction (no wheal) probably because the wheal was small. However, the 3D imaging system has a high depth-sensitivity, and the wheals are correctly detected. It is not uncommon for false-positive reactions to appear at the site of the negative control due to the trauma that the pricking device imparts on the skin [38]. Moreover, we are certain of the detected wheals due to the regularly distributed prick test sites provided by the multi-test device, as shown in Fig. 1(A). The measurement discrepancies may be due to the ruler resolution and irregular wheal shapes that by visual inspection could lead to significant variability.

The work is promising, but I encourage the authors to work in these two points before publishing it.

RE: Thank you for your suggestions. They have allowed us to improve our manuscript.

Reviewer #2:

In the manuscript, the authors present an excellent report on the performance of a new 3D imaging system they have developed for automated parameterizing of wheals caused by allergic reactions during the skin prick test commonly used in medicine. They go in considerable detail to explain nearly every aspect of its construction and operation, including the underlying mathematical operations that automatically transform a clinical image of a patients skin into wheal diameter information. They include a clinical study in which they compare the performance of their system to standard methods of assessing allergic reactions as used by medical specialists in diagnoses and convincingly show a very promising potential of the new method.

There are, in my opinion, a few minor details that, when resolved, would improve the presentational value and consistency of the manuscript. They are (in the order as they appear in the manuscript):

1. In the Introduction section, the authors might be interested in the paper (Laloš, Mrak, Pavlovčič, Jezeršek, Handheld optical system for skin topography measurement using Fourier transform profilometry, Journal of Mechanical Engineering 61(5), pp. 285-291 (2015), doi: 10.5545/sv-jme.2015.2424) in which similar pattern projection and triangulation methods have been used to determine the skin surface topography.

RE: Thank you for your recommendation of this reference. We reviewed it and concluded that although it is similar in the in terms of 3D surface recovery via fringe projection profilometry, their device is designed for a different purpose than ours and could not be readily used for assessing the SPT. In particular, their field of view (FOV) is quite small. This situation is similar to the pioneering work by dos Santos et al., 2008 in which they were able to measure only on wheal within the FOV. Nevertheless, we have cited this reference, and we have also included two additional references in the introduction — first, a recent survey on 3D scanning applications in the medical field [16]. And second, a recent conference presentation of a feasibility study on the use of 3D scanners for skin prick allergy testing [21]. We have modified this part of the introduction in the following way:

3D scanning technology is becoming an essential tool in many medical subfields [16]. It has been applied for the monitoring of skin treatment therapy [17], or the characterization of cancer lesions [18]. However, each procedure or application has specific metrological requirements, like the field of view or the depth resolution, which typically render a device unsuitable for multiple applications. We know of three earlier works which make use of 3D technology for assessing the SPT [19–21]. The work of dos Santos et al. [20] was the first to show the advantages of 3D imaging technology for measuring wheals. Although, they used a commercial 3D scanning device [22,23] with a relatively small field of view (FOV) in the order of 40 × 30 mm, which could only register one wheal at a time. This limitation impedes its use in the typical clinical setting where many SPTs are typically applied simultaneously. In a feasibility study by Verdaasdonk et al., the authors used general-purpose 3D scanners for assessing the SPT. They tested the scanners extensively on phantom objects, like buttons, but provided few clinical data (17 positive allergic reactions) which were assigned a reaction grade 1, 2, or 3 by a dermatologist instead of validating with a more quantitative approach. Also, they did not discuss any automated detection and measurement procedure.

2. In the Contribution section, lines 79 and 80, the authors successively reference Fig. 1 and Fig. 3. It is unusual to skip a number when first referencing figures. They might consider referencing Fig. 2 in between the two to maintain the proper figure numbering.

RE: Thank you for pointing this out. We have included the following sentence in between referencing Fig. 2, which in the new version of the manuscript is now Fig. 3:

In this work, we use an in-house developed wide-field 3D imaging system [19], shown in Fig. 2, for the 3D reconstruction of the SPT, which we validate with objects of known shape shown in Fig. 3. We propose a novel method for the robust automated measurement of the skin wheals. Our proposed method, depicted in Fig. 4, is based on advanced 3D data processing.

3. In Tables 1 and 2, the authors use Greek mu and sigma signs to denote average and standard deviation, respectively. While this is a common practice, explicitly stating what these symbols represent, by adding them into text in lines 130 and 131, for example, would preserve the consistency of the manuscript.

RE: Thank you for this suggestion. We have changed the Greek letters with the more standard letters x bar (\bar{x}) for the estimated mean value and s for the estimated standard deviation. We updated the Table and have modified the suggested lines with the following:

In Table 1, we report the values of the average diameter \bar{x} and the standard deviation s of the measurements for each spherical cap obtained for camera 1 and camera 2. The measurements are accurate within ±0.1 mm.

4. It would be most illuminating if the authors could add an unaltered image of the patients forearm, such as what a physician would normally see during his evaluation, to Fig. 4 so that it could be compared to their images denoted S and S'. Provided such an image exists at all.

RE: Thank you for your recommendation, which improves the interpretability of the figures.

We have now included a new figure (Fig. 1) in which we describe the skin prick test procedure and its assessment by the physician. We believe this figure improves the manuscript. Following your suggestion, we have included the texture image in Fig. 6 (Fig. 5 in the previous manuscript) to emphasize the correct detection of the wheals. The image has been digitally sharpened to improve the visualization of the wheals in the skin. The new figure with the modified caption is the following:

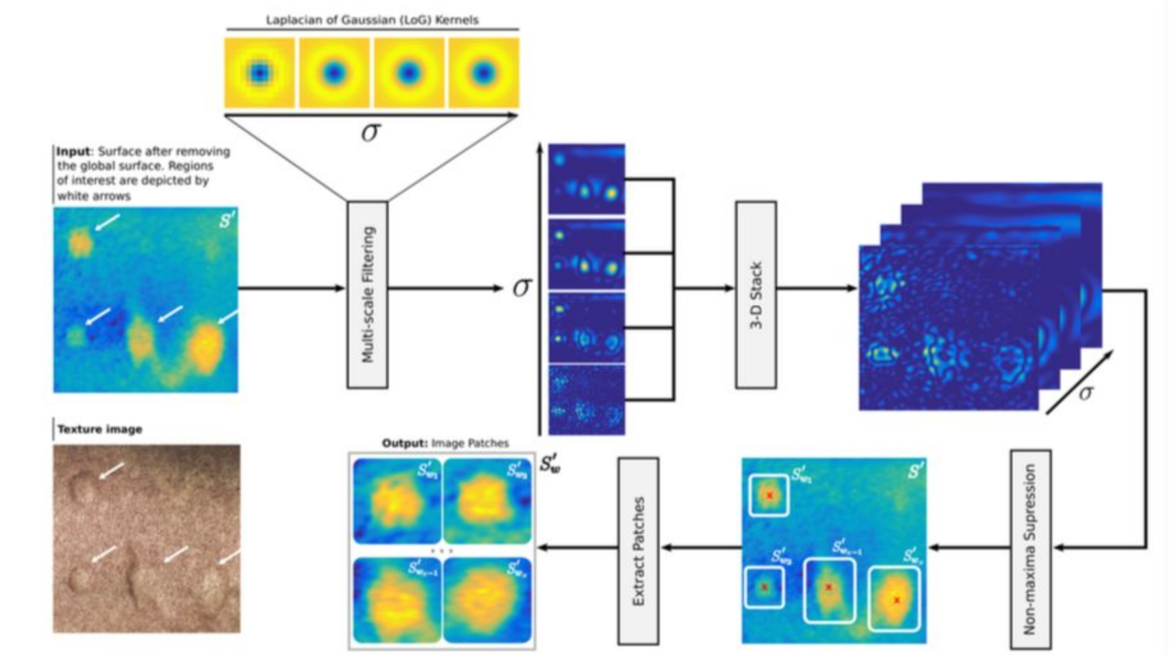

Fig 6. The *Wheal Detection* stage. S' and S'_w denote the surface after removing the global curvature of the forearm and the extracted surface patches, respectively. We have included the texture image to highlight the correct detection of the wheals. This image has been digitally sharpened to improve the visualization of the wheals.

5. In Eq. (1), the left curly bracket is used very unusually. It might be more elegant to separate the equations and give each of them their own number.

RE: Thank you for your comment. Reviewer 1 had several concerns regarding the parametric model; please refer to answer 1 of reviewer #1. However, since we have maintained this model in the revised version of the manuscript, we have now changed the way we mathematically describe the models.

First, we have explained in more detail the parametric fitting of any model to our 3D data in three general steps. We have decoupled the rotation via a change of variables from the parametric model equation. The rotation part of the model is now in equation (1), regardless of the parametric model. Second, the parametric model equation for the super-Gaussian is now explicitly defined in equation (4).

6. It is not apparent how the rotational angle theta in the second part of Eq. (1) and in the line 190 is obtained. It would be good to explain this.

RE: Thank you for this suggestion. We have addressed this issue in the previous answer. However, we have improved our description of what the theta angle represents and how it is obtained iteratively with the Trust region reflective non-linear least squares algorithm. We have also added a new citation to the reference of this algorithm.

7. It might be an informative duscussion to briefly compare the times needed to arrive to the wheal diameters by the new metod with those needed to obtain such diameters by standard manual methods.

RE: Thank you for pointing out a feature that may interest many readers. We have included a final subsection in the Results called Automated SPT assessment time in which we give an overall value of the time our system takes to scan and produce the wheal measurements. In the example that we used for illustrating the method, our system takes around 16 seconds, compared to an average of 1.5 minutes the physician takes to produce the measurements. However, we are cautious in stating that further research in a clinical setting is needed. We have included the following in the manuscript:

Automated SPT assessment time

The total runtime of the automated reading of the SPT depends on the number of wheal reactions. However, to give the reader an idea of the time the implemented system takes to scan and process the 3D data, we report here the time it took for the example shown in Fig. 4. In this case, the subject's arm had four wheals. Our system was controlled via a PC with Windows 7 (2.4 GHz i7 intel processor, 8 GB RAM) and MATLAB R2017. The overall processing includes three stages: acquisition, 3D reconstruction, and robust diameter estimation. The acquisition of the images takes around 500 ms. This time is the same for all acquisitions regardless of the number of wheals. This stage is the only one in which the subject has to interact with the device. The 3D reconstruction stage includes the fringe image processing via Fourier Transform Profilometry and the phase-to-metric coordinate mapping. This stage takes around 1.5 s. Finally, the robust estimation of the wheal diameters requires about 14 s. This stage includes the pyramidal decomposition of the 3D reconstruction (~ 0.061 s), the wheal detection(~ 4.56 s), and the parametric fitting (~ 9.48 s). The total runtime was 16 s. While this time could be further improved with parallel 421 computing, we believe it is a reasonable processing time compared to 1.5 minutes on average that the physician takes to manually assess the SPT. However, further research is needed in a clinical setting to assess this aspect correctly. Moreover, if the physician requires a permanent record of the test, a traced copy of the wheals takes several minutes to produce. Nevertheless, the main purpose of our approach is to have a digital record of the test with accurate and reliable wheal measurements.

In view of this, I do recommend the manuscript entitled 'Robust automated reading of the skin prick test via 3D imaging and parametric surface fitting' to be published in the PLOS ONE journal with minor revisions.

RE: Thank you for your suggestions and overall constructive review.
